# Attachment buffers the physiological impact of social exclusion

**DOI:** 10.1371/journal.pone.0203287

**Published:** 2018-09-05

**Authors:** Belinda J. Liddell, Bronte S. Courtney

**Affiliations:** School of Psychology, UNSW Sydney, Sydney, NSW, Australia; Technion Israel Institute of Technology, ISRAEL

## Abstract

Attachment systems facilitate coping with stress, with previous studies demonstrating attachment figures diminishing subjective, behavioral and neural responses to social pain. Yet little is known about the physiological mechanisms governing this benefit in the context of social exclusion. This study investigated the impact of attachment (vs non-attachment) priming on affective and cardiovascular responses to social exclusion induced by the computerized “Cyberball” ball-tossing game, and the moderating influence of individual differences in attachment style, rejection sensitivity and self-construal. No significant change in high frequency heart rate variability (HF-HRV)–an index of parasympathetic activity and cardiovagal balance–was observed across the time course in the attachment priming condition, whereas the non-attachment condition showed significant fluctuation in HF-HRV–increasing during Cyberball and decreasing relative to baseline during recovery. Moreover, the benefit afforded by attachment priming on was enhanced amongst participants with lower rejection sensitivity and higher collectivistic self-construal, and those with higher anxious attachment style in the non-attachment prime group showed a trend towards increased HF-HRV during the Cyberball. Results are consistent with Social Baseline Theory, which argues that social proximity–particularly from attachment figures–protects against the metabolic costs associated with strong reactions to stress, including the preservation of cardiovagal homeostasis in this instance. Social attachments may provide an important mechanism to increase adaptive responding to the distressing experience of social exclusion.

## Introduction

Human attachment has evolved as a behavioral system that binds infants to their primary caregivers [1], and in adulthood, the internal activation of attachment figures continue to assist coping with challenging situations [2]. Attachment theory is supported by behavioral studies demonstrating that subliminal threat priming increases cognitive accessibility of attachment-related words [3]. The emerging Social Baseline Theory argues that close relationships and proximity to others encourages the social regulation of emotion, thus conserving vital metabolic resources for other processes such as executive functioning [4]. An increasing number of studies support this notion: explicit or pictorial presence of a romantic partner during exposure to stressful, painful or aversive events reduces salivary cortisol responses [5], diminishes the subjective feeling of pain [6] and decreases activity in threat detection [7] and pain regions of the brain such as the dorsal anterior cingulate (dACC) [6]. Attachment figures have also been shown to strengthen engagement of emotion regulation systems, namely the ventromedial prefrontal cortex [6]. Despite evidence supporting the role of attachment figures in responding to threat, little is known regarding the physiological mechanisms governing this benefit, nor the possible contributory role of individual differences in core factors such as attachment style, rejection sensitivity or individualistic-collectivistic self-construal. This study will assess whether such factors moderate the hypothesized buffering effect of attachment figures on cardiovascular responses to social exclusion.

Social exclusion has acute psychological and physiological effects, arguably because it interferes with four fundamental human needs: belonging, self-identity, control and to live a meaningful existence [8]. Social exclusion can increase salivary cortisol [9], change heart rate [10] and increase engagement of dACC suggesting enhanced ‘social pain’ [11], an effect which may be mitigated by social support [12]. Adaptive responses to demanding situations require effective emotion regulation, whereby the individual adjusts to conditions of heightened arousal and selects situationally appropriate behavioral responses. An important physiological mechanism underpinning emotion and social regulation is heart rate variability (HRV)–an index of beat-to-beat variations in heart rate [13]. According to Polyvagal Theory, threat exposure accelerates heart rate and decreases HRV via unmyelinated dorsal vagal innervation over the heart [14]–a state associated with stress, psychopathology and physical disease [15]. Conversely, during times of relative safety, the parasympathetic nervous system dominates via the myelinated ventral vagal complex, ensuring adaptive prosocial regulatory behavior including social engagement [16]. This is reflected in increased HRV, indicative of efficient emotion and self-regulation processes [17]. Indeed, healthy vagus nerve functioning has been argued as a central mechanism that links social connection with others to overall health and wellbeing [18]. Thus, we suggest that if attachment is a socially adaptive mechanism that assists in regulating adverse emotional responses to exclusion, then this process is likely to be reflected in increases in HRV during and post-rejection.

The benefit afforded by attachment during social exclusion may be moderated by a number of individual differences, but the role of individual differences have not been explored to date. One core individual difference known to influence the impact of attachment priming is attachment style. Attentive caregiving during infancy promotes a secure attachment style, whereas adverse caregiving can produce insecure attachment styles that vary along two dimensions–anxious or avoidant insecurity [1]. Anxious attachment style is characterized by an exaggerated need for closeness with others, whereas avoidant attachment style is marked by excessive independence and emotional distance in relationships [19]. Both insecure styles are considered maladaptive, demonstrating low HRV and enhanced threat-related processing [20]. Moreover, those with insecure styles may be less able to draw benefit from attachment cues during stress. For instance, attachment priming enhances affective recovery following autobiographical recall of upsetting events [21] and reduced trauma memories [22], but only amongst participants low in avoidant attachment style. However, it is unclear how insecure attachment style may moderate the effect of attachment priming on the experience of social exclusion.

Other individual differences may also contribute to the effects of feeling excluded. We have selected two additional mechanisms to examine in the current study. The first is rejection sensitivity–a dispositional trait marked by a heightened tendency to expect, perceive and respond strongly to rejection [23, 24]. Studies have found that people high in rejection sensitivity report greater depression symptoms [25], hostility [26] and aggression [24, 27] following peer rejection. Cognitively, rejection-related words have been shown to exert stronger levels of interference in those more sensitive to rejection [23]. Importantly, rejection sensitivity has been associated with heightened physiological arousal and lower HRV during social exclusion or threat exposure [27]. Therefore, we argue that rejection sensitivity may also moderate attachment influences over subjective and cardiovascular responses to social pain.

Self-construal is often over-looked in attachment literature. It defines how one views the self in relation to others [28], and is characterized by the individualistic-collectivistic dichotomy: individualists value an independent self–with a focus on the ‘I’, whereas collectivists hold a self-perspective that is interdependent with others, with a focus on the ‘we’. Several studies suggest that self-construal might moderate how social exclusion is experienced. For example, those with a collectivistic self-construal evidenced enhanced recovery following social exclusion compared to those with an individualistic self-construal, possibly reflecting enhanced cognitive accessibility to implicit social support resources [29]. A recent study showed an interaction between self-construal and attachment style, such that only avoidant individualistic (and not collectivistic) participants were less distressed by social exclusion [30]. Thus, it appears that attachment style, self-construal and rejection sensitivity are important factors to consider when examining how attachment may buffer the negative physiological effects of social exclusion.

This study aims to examine whether mental representations of an attachment figure activated via priming (vs non-attachment control) attenuates the effects of social exclusion on cardiovascular responses and change in subjective affect, and whether these effects are moderated by attachment style, rejection sensitivity or self-construal. If attachment priming successfully buffers the impact of social exclusion, we expected to observe heightened HRV and lowered heart rate responses during the reactive exclusion phase, followed by an expeditious return to baseline HRV during recovery consistent with Social Baseline Theory [4], relative to the non-attachment control condition. It was also predicted that the advantages offered by attachment priming would be diminished in those with higher avoidant attachment style and rejection sensitivity, as well as enhanced amongst those with higher collectivistic self-construal.

## Materials and methods

### Participants

One hundred healthy undergraduate psychology participants took part in return for course credit. Eight participants were excluded from the sample following the experiment due to self-reported psychoactive substance use (n = 2), use of medication capable of altering cardiovascular activity (n = 3), self-reported diagnosis of a psychological disorder (n = 1) or insufficient electrocardiogram (ECG) recordings (n = 2). The final sample comprised N = 92 participants (36 males, 56 females, mean age = 19.8 years (SD = 2.13), age range 18–29 years). Participants were randomly assigned into either the attachment (N = 47) or non-attachment (N = 45) condition. The full demographics of the sample is provided in Table 1. Sample size was determined before analysis.

**Table 1:** Participant characteristics.

	Attachment Prime *(n = 47)*	Non-Attachment Prime *(n = 45)*	
	M (SD)	M (SD)	t (*p*)
Age	19.91 (2.29)	19.76 (1.98)	.36 (.72)
Anxiety Attachment Score	2.51 (.61)	2.37 (.60)	1.13 (.26)
Avoidant Attachment Score	1.83 (.81)	1.74 (.78)	.49 (.63)
Individualistic Score	55.64 (7.91)	55.78 (10.72)	-1.09 (.28)
Collectivistic Score	57.64 (6.98)	57.29 (9.63)	.20 (.84)
Rejection Sensitivity	9.58 (3.44)	8.60 (3.04)	1.45 (.15)
Imaginal capacity	34.15 (7.17)	37.09 (8.24)	-1.83 (.07)
Physical activity (Total)	5668 (3650)	4744 (3031)	1.32 (.19)
Gender distribution	***n***	***n***	**χ**^**2**^ ***(p)***
Males	17	19	
Females	30	26	.35 (.55)
Smoking status			
Current smoker	1	5	
Not a current smoker	46	40	3.04 (.11)*
Alcohol consumption: Frequency			
Never	12	9	
Monthly	24	14	
Weekly	8	16	
Some days each week	3	5	
Most days each week	0	1	7.19 (.13)
Caffeine consumption			
Never/Less than once a month	18	7	
Less than once a week	7	9	
Less than once a day	14	15	
At least once a day	8	11	3.00 (.39)

*Fisher’s exact test used for smoking status as one cell has a frequency of less than five.

### Materials

Information regarding variables known to influence cardiovascular responses was obtained, including smoking status, psychoactive substance use, alcohol consumption (AUDIT-C) [31], presence of cardiovascular and other medical conditions, caffeine consumption via the Melbourne Food Frequency Questionnaire [32], medication use and physical exercise via the International Physical Activity Questionnaire [33].

Attachment style was measured via the Experiences in Close Relationships Questionnaire–Revised (ECR-R) [34]. Responses are provided on a 7-point Likert scale (ranging 1 = strongly disagree; 7 = strongly agree), yielding total scores on two subscales–attachment anxiety (Cronbach α = 0.87) and attachment avoidance dimensions (Cronbach α = 0.94).

Rejection sensitivity was indexed by the short-form Rejection Sensitivity Questionnaire (RSQ), which presents participants with eight hypothetical, rejection relevant scenarios that are rated on their degree of concern (anxiety score) and expectations of acceptance (expectancy score) [35]. A rejection sensitivity score was obtained for each situation by reversing the expectancy score and multiplying it by the anxiety score. A total rejection sensitivity score was generated by computing the mean of the eight situation scores (Cronbach α = 0.72).

Self-construal was indexed via the self-construal scale [36], comprising 12 individualistic and 12 collectivistic statements, with participants rating the degree to which they agree or disagree with the statement on a 7-point Likert scale. Separate scores for individualistic (Cronbach α = 0.79) and collectivistic sub-scales (Cronbach α = 0.70) were computed, along with a self-construal index to determine predominant self-construal (subtracting sum of collectivistic items from sum of individualistic items).

Imagination capacity was tested via the Vividness of Visual Imagery Questionnaire (VVIQ), as the experimental manipulation involved the mental representation of a specific person (Marks 1973), with average visualization scores computed (Cronbach α = 0.81). This variable was included to check for any significant group differences in imagination capacity, rather than as a specific moderating variable effecting the impact of attachment priming on objective and subjective measures.

Explicit mood was measured by the Positive and Negative Affect Scales (PANAS) [37], which was administered both prior to and following recovery from exclusion. The 20 item scale consist of 10 positive and 10 negative mood descriptors and participants rated the extent to which they presently felt each mood state on a 5-point Likert scale (ranging 1 = not at all/very slightly to 5 = extremely). Separate positive and negative scores were calculated and had good internal consistency (negative time 1: Cronbach α = 0.85; negative time 2: Cronbach α = 0.85; positive time 1: Cronbach α = 0.86; positive time 2: Cronbach α = 0.91). All measures were administered electronically using MediaLab (V.2012) software.

### Design

#### Social exclusion

Social exclusion was experimentally induced using the 4-player version of the ‘Cyberball’ task–a computerized ball-tossing simulation between the participant and three programmed confederates [38], and which has been demonstrated to robustly induce the subjective perception of exclusion [8, 11, 39]. Prior to commencing the Cyberball task, a webcam and Adobe Photoshop (v. CS6) was used to insert a photo of the participant as ‘Player 2’ on the screen during play. During the game, participants threw the ball to their intended recipient by clicking on their photograph with the mouse. The game was programmed so that the during the initial inclusion phase, the participant had an equal chance of receiving the ball (in total twice from each player, lasting 30–45 seconds). This was followed by the exclusion phase whereby participants were excluded from all play for the remainder of the 5 minute Cyberball game. Pilot testing indicated that participants found it difficult to maintain attention on the game during the exclusion phase (based on self-report and observations of reduced concentration). To reduce the decline in attentional focus, participants received the ball once again towards the end of the game to maintain attention. The game lasted for a total five minutes, equating to approximately 40 throws in total.

The 20 item ‘Need-Threat Scale (NTS)’ [40]–adapted from Williams (2009)–was administered as a manipulation check to confirm whether the Cyberball successfully induced exclusion and to detect participant scepticism about the authenticity of the game (participants reported the percentage of throws they believed they received). The scale was also used to assess the subjective impact of ostracism by measuring satisfaction levels on four fundamental needs. Using a 5-point Likert scale (1 = *not at all*, 5 = *extremely*), participants rated 20 statements on the degree to which they represented their feelings during the task. Internal consistency was high (α = 0.85). The items targeted the fundamental needs of belonging (e.g. ‘*I felt I belonged to the group*), self-esteem (e.g. ‘*I felt good about myself’*), meaningful existence (e.g. ‘*I felt useful’*) and control (e.g. ‘*I felt powerful’*). A needs-satisfaction score was computed by summing scores of items within each of the four needs to determine if there was a priming effect.

### Procedure

Participants were instructed to abstain from caffeine consumption for four hours prior to completing the study. Upon arrival at the laboratory, participants were told they would be taking part in a study on mental visualization, and written informed consent was obtained in accordance with ethics approval from the UNSW Sydney Human Research Ethics Approval Panel (HREAP). Participants then completed the self-report measures listed above, whilst the ECG electrodes were attached and their photograph taken ready for Cyberball participation. The screen was then switched off, and participants were instructed to relax in a seated position for five minutes; this comprised the baseline recording.

Participants were randomly allocated to either the attachment or non-attachment priming condition. In the attachment priming condition, participants were asked to nominate a person they spend a lot of time with, would miss if separated for a prolonged period and rely upon for support in stressful situations. For the non-attachment priming condition, participants were directed to consider an acquaintance–someone they know and interact with, but are not particularly close to. These directions were derived from the “WHOTO” scale used to identify attachment figures [41]. Participants then mentally activated this figure by verbally responding to three questions (‘Who is this person and what is their relation to you?’; ‘Describe this person’s personality’ and ‘Describe your typical interaction with this person’). Finally, participants rated their perceived closeness with their nominated figure on a scale from 0 (not close at all) to 10 (extremely close). They were then instructed to visualize the nominated person for a further two minutes, focusing on their typical interactions and personal mannerisms, with no interference from the experimenter.

Participants were told they were to play ‘Cyberball’–a game for mental visualisation–with three other players. They were presented with instructions for playing the game on the screen, before entering their name and proceeding to play. After the completion of Cyberball, participants were instructed to sit still for a further five minutes (recovery phase), following the same protocol as baseline recording. The experimenter then removed the electrodes. Each experimental phase was allocated five minutes, as this is the minimum acceptable time to accurately measure short-term resting-state HRV [42]. Finally, participants completed a second PANAS and the NTS, and were fully debriefed.

#### Heart rate data collection

Cardiovascular measures were measured by Powerlab (ADInstruments), using disposable electrocardiogram (ECG) electrodes placed bilaterally under the clavicle, with the ground electrode attached to the vertebra prominens (C7) on the back of the neck following skin preparation using a gentle abrasive gel. Participants were instructed to minimize movement during recording. Labchart software (v.18) continuously recorded ECG during baseline, exclusion and recovery phases of the experiment.

### Data analysis

Heart rate (HR) and HRV data was considered across three time periods, each of 5 minutes duration: baseline, the Cyberball reactivity phase (i.e. encompassing initial inclusion for 30–45 seconds and subsequent exclusion), recovery phase following the Cyberball. HR and HRV data were preprocessed and analyzed using Labchart software, which applies adjustable algorithms to detect R-peaks in the QRS complex of the ECG. Scoring was manually checked and adjusted where necessary, including the exclusion of movement artefact from the data. HR was computed as the number of beats per minute (bpm). High frequency power (in its absolute and normalised form) was the primary HRV variable interest (HF-HRV) as it is most closely associated with parasympathetic modulation of the autonomic nervous system [43]. High frequency power in normalised units is a more stable index of HRV when there is a high degree of variation (i.e. standard deviation) in the absolute data [42], as was apparent in the current data set, and thus we report both indices here (high frequency absolute (HF(abs) and high frequency normalised units (HF(nu))). A fast Fourier transform (FFT) algorithm was used to determine HF-HRV frequency data with a window of 0.15–0.4 Hz, and ectopic beats were excluded from HRV analyses.

HR/HF-HRV metrics were natural log-transformed due to abnormal distributions (with the exception of normalised units). Transformed data were normally distributed and screened for outliers, with replacements made with integers reflecting 3 standard deviations (+/-) from the mean (0.02% of total transformed data). To account for individual differences in baseline recordings of HR and HF, differences scores were computed from the log-transformed data for the two phases of interest. The first phase related to the Cyberball reactivity phase (cyberball–baseline), and the second examined how HR and HF-HRV returned to baseline during the recovery phase to test hypotheses in line with social baseline theory (i.e. recovery–baseline). HR and HF-HRV difference scores were used in all analyses.

We conducted two sets of analyses with these dependent variables. First, in order to examine the effect of the attachment prime on both subjective (PANAS ratings) and objective responses (heart rate, HF-HRV) to social exclusion, we conducted mixed-model ANOVAs with the between-group factor being attachment (vs non-attachment) prime group, and the within-subject factor being phase of social exclusion (cyberball reactivity, recovery); alpha level p < .05, with posthoc tests being Bonferroni-corrected.

Second, to examine the role of individual differences, hierarchical moderated regression analyses were conducted to assess the relative contribution of attachment style, rejection sensitivity and self-construal as predictors to change in HR and HF-HRV during cyberball reactivity and recovery phases, as well as change in mood state, between groups. Predictors at the first step comprised of attachment prime condition. Independent predictor variables were initially grand mean centered prior to analyses to correct for multicollinearity. Moderation variables were avoidant attachment style, anxious attachment style, rejection sensitivity, individualistic self-construal and collectivistic self-construal scores, each which were entered into the model at the second step. Interaction terms were generated by multiplying centered variables with attachment prime condition, which were then entered into the model at the third step. Final regression models were then computed retaining only the important predictors (p < .05). Simple slopes analyses were conducted to examine significant interaction terms, with slopes significant if p < .025, Bonferroni corrected for testing two slopes per interaction.

The aggregated data used in the analysis is presented the accompanying in S1 Dataset.

## Results and discussion

### Participant characteristics

Table 1 presents demographic and self-report data for the two prime groups. Independent samples t-tests revealed no statistically significant group differences in age, attachment style, self-construal index or levels of individualistic or collectivistic self-construal, rejection sensitivity or imaginary capacity (p > .05). Chi-square also indicated no significant differences in the distribution of sex or cultural backgrounds between groups. The overall sample comprised equal numbers of males (n = 36; 39.1%) and females (n = 56, 60.9%), and while 71.7% (n = 66) participants were born in Australia, the remaining participants were from a range of cultural backgrounds (38.3%; including Hong Kong, Bosnia, China, India, Japan, South Africa, Singapore and the United States), ensuring this was a diverse sample.

In order to verify any observable group differences in heart rate measures could be attributed to the experimental manipulation, other factors known to influence cardiovascular activity were measured. There were no significant group differences in frequency of alcohol or caffeine consumption, smoking status, or level of regular physical activity (p > .05; see Table 1).

### Manipulation checks

Mean estimates for the percentage of throws received by the participant were M = 13.2% (SD = 5.6) and M = 13.5% (SD = 4.4) for the attachment and non-attachment prime groups respectively. This is below chance in both groups (25% constitutes equal distribution among four players), indicating that exclusion was successfully induced. The majority of the sample indicated they felt at least moderately ignored or excluded based on a 5-point rating system (see Table 2), and there were no discernible difference between groups. Moreover, no significant differences in regards to responses on the need-threat scale between attachment prime group were observed (Table 2), such that attachment priming was found to have no significant impact on any of the four fundamental needs (p > .05).

**Table 2:** Need-threat scale responses.

	Attachment Prime *(n = 47)*	Non-Attachment Prime *(n = 45)*	
	M (SD)	M (SD)	t (*p*)
Need-Threat Scale responses			
Belonging	13.85 (4.11)	14.24 (3.36)	-.50 (.62)
Self-esteem	13.98 (3.12)	13.78 (3.37)	.30 (.77)
Meaningful existence	15.09 (3.82)	15.33 (3.18)	-.34 (.74)
Control	10.32 (2.40)	10.42 (2.73)	-.19 (.85)
Feeling ignored	3.13 (1.08)	3.07 (1.12)	.27 (.79)
Feeling excluded	3.26 (1.03)	2.98 (1.08)	1.26 (.21)

The attachment prime group rated their level of closeness with their nominated figure as higher than the non-attachment prime group (*t*(90) = 15.64, *p* < .001), with a large effect size (Cohen’s *d* = 3.24). As directed in the instructions, the most common relationship categories nominated were ‘*parent’* for the attachment prime condition (38.3%) reflecting strong social attachments, followed by ‘*romantic partner*’ (27.7%), ‘*close friend*’ (21.8%) or ‘*sibling*’ (12.2%). In the non-attachment prime condition, the largest category nominated was ‘*classmate’* (28.9%), followed by ‘*friend of a friend’* (24.4%), ‘*colleague*’ (15.6%), ‘*old friend*’ (13.3%), ‘neighbor’ (8.9%), ‘*distant relative’* (6.7%), and ‘*coach*’ (2.2%), with each category representing an acquaintance.

### The effect of attachment priming on HR and HF-HRV

**Fig 1:**
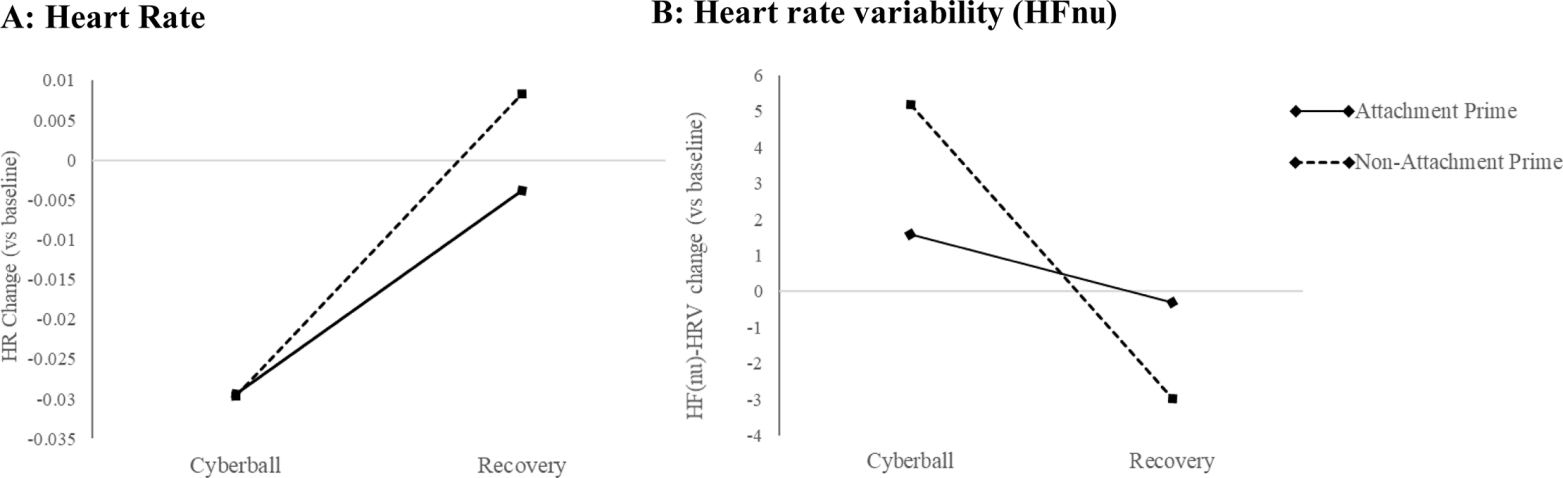
Heart rate and HRV changes in Cyberball and recovery phases. 1A: Heart rate (beats per minute) change relative to baseline; 1B: Heart rate variability (HFnu-HRV) change relative to baseline in Cyberball and Recovery phases (x-axis) in Attachment and Non-Attachment Prime Groups. Baseline is represented by the 0 on the y-axis in both figures.

For HR, a significant main effect of phase was observed (F(1,92) = 26.25, p < .001, η_p_^2^ = .23), whereby HR was significantly lower during the reactivity relative to recovery period across both groups relative to baseline (noting again, that difference scores are used for reactivity and recovery phases; see Fig 1A). No significant interaction effect with condition was observed for HR.

A 2 (Condition: attachment vs non-attachment prime) x 2 (Phase: Cyberball reactivity vs recovery) repeated-measured ANOVA revealed a significant condition by phase interaction for HF(nu): F(1,90) = 5.74, p = .019, partial eta squared η_p_^2^ = .06. Post hoc pairwise comparisons revealed that there was a significant difference between the cyberball reactivity and recovery phases in the non-attachment prime group (p < .001 Bonferroni corrected; 95% C.I. 0.11–0.33). This effect was such that HF(nu) increased relative to baseline in the reactivity phase, but significantly decreased relative to baseline in the recovery phase for those specifically in the non-attachment prime condition (Fig 1B). No significant difference was observed across phases compared to baseline in the attachment prime condition (p = .54), thus remaining relatively stable across the time course of the experiment. No significant effects were observed for HF(abs), which may be attributed to the large standard deviations in the HF(abs) data [42].

### The effect of attachment priming on subjective affect

ANOVAS conducted to investigate the effect of condition on change in mood state revealed a significant time effect for positive (F(1,90) = 39.41, p < .001, η_p_^2^ = .31) and negative mood (F(1,90) = 8.13, p = .005, η_p_^2^ = .08), whereby both positive and negative mood decreased over the course of the study. No group differences were observed between prime group (p >.05).

### Regression analyses

Regression analyses were conducted to examine the contributing role of attachment style, rejection sensitivity and self-construal in moderating the impact of attachment prime on change in HR (reactivity and recovery), HF-HRV (reactivity and recovery, HF(nu) only) and subjective affect (positive and negative). Initial models with all variables were initially conducted, with final models retaining significant coefficients described below (full initial and final models are provided in S1 File). In all models, multicollinearity was not a concern (tolerance scores for all predicting variables exceeded 2.7, VIF scores < 2.5) and errors were sufficiently independent (Durbin-Watson value was around 1.7 for each analysis).

#### Heart rate

**Fig 2:**
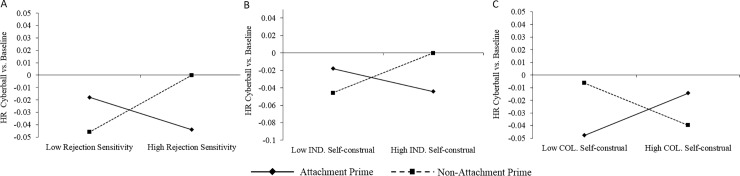
Simple slopes analyses for heart rate changes relative to baseline during Cyberball. (2A). Rejection Sensitivity; (2B) Individualistic (IND) Self-construal; (2C) Collectivistic (COL) Self-construal.

We found there to be several factors moderating the influence of the attachment prime group on heart rate reactivity during Cyberball, with the final model being significant at the third step (F(7, 84) = 2.91, p = .01, R^2^ = .20); see Fig 2.

First, rejection sensitivity interacted with attachment prime condition (B = .01, t = 3.00, p = .004). Simple slopes analysis revealed that for participants in the non-attachment prime group, those who were low in rejection sensitivity displayed decreased HR during Cyberball relative to baseline, whereas HR did not change in those more highly rejection sensitive (B = .01, t = 2.30, p = .02); the attachment prime group slope was not significant (p = .092; Fig 2A).

Second, individualistic self-construal interacted with attachment prime condition (B = .005, t = 3.44, p = .001). Simple slopes analysis showed that for those in the attachment prime group, high individualistic self-construal predicted a greater decline in HR during Cyberball (B = -.003, t = -.302, p = .003); Fig 2B. The slope for the non-attachment prime group was not significant at corrected levels (B = .002, t = 1.99, p = .05), but the trend showed the opposite effect to the attachment prime, whereby high individualistic self-construal was associated with a greater decrease in HR during Cyberball compared to low individualistic self-construal.

Finally, an interaction between collectivistic self-construal and prime group was observed (B = -.004, t = -2.68, p = .009), notably in the opposite direction to individualism. For participants in the attachment prime group, a trend effect showed that those high in collectivistic self-construal evidenced less HR deceleration during Cyberball then those low in collectivistic self-construal (B = .002, t = 2.00, p = .049); Fig 2C. For participants in the non-attachment prime group however, high collectivistic self-construal predicted greater deceleration in HR compared to low collectivistic self-construal (B = -.002, t = -2.28, p = .025). No significant predictors were observed for HR during the recovery phase.

#### HF-HRV

**Fig 3:**
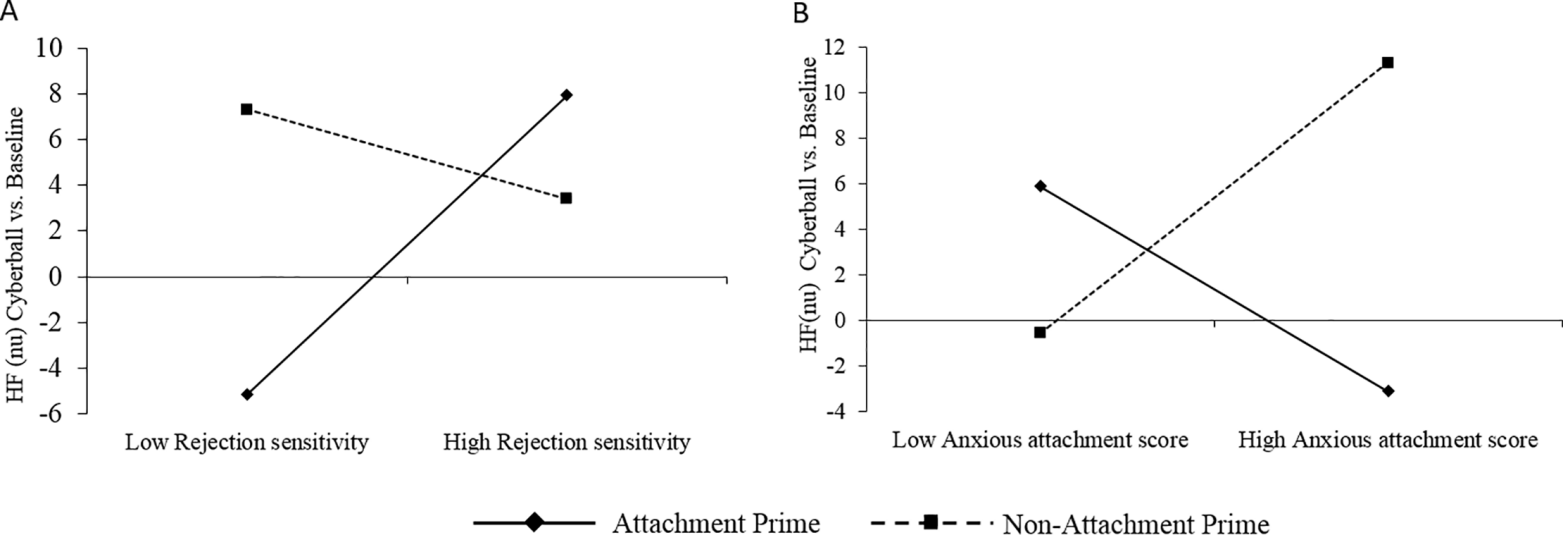
Simple slopes analysis for HF-HRV changes relative to baseline during Cyberball. (3A). Rejection Sensitivity; (3B) Anxious attachment.

The final model predicting HF (nu) change during the Cyberball reactivity period was of trend significance (F(6,85) = 2.12, p = .059, R^2^ = .13); Fig 3.

Rejection sensitivity was a significant predictor (B = 2.00, t = 2.55, p = .012), as well as in interaction with attachment prime condition (B = -2.60, t = -2.07, p = .041). Simple slopes analyses revealed that the attachment prime increased HF-HRV in those more highly rejection sensitive, but decreased HF-HRV in those with low rejection sensitivity (B = 2.00, t = 2.55, p = .01); no difference was observed for the non-attachment prime group (B = -.6, t = -.59, p = .56), Fig 3A.

A second interaction effect was observed between anxious attachment style and attachment prime condition (B = 17.29, t = 2.66, p = .009). For participants in the attachment prime group, anxious attachment style did not moderate change in HF-HRV during Cyberball (B = -7.46, t = -1.63, p = .12); Fig 3B. For those in the non-attachment prime group, there was a trend towards high anxiously attached individuals showing increased HF-HRV during Cyberball, which was not evident in those with lower anxious attachment (B = 9.83, t = 2.02, p = .046). Again, there were no significant predictors of HF-HRV during the recovery phase.

#### Subjective affect

When predicting change in positive mood, significant predictors included individualistic self-construal (B = .02, t = 3.36, p = .001) and avoidant attachment style in interaction with attachment prime condition (B = .39, t = 3.19, p = .002; Final model F(4,87) = 6.04, p < .001, R^2^ = .22).

Simple slopes analysis revealed that those higher in avoidant attachment style showed *less* decrease in positive mood over the course of the study than those lower in avoidant attachment style in general, but particularly those in the non-attachment prime group–where positive mood did not fluctuate (slope for attachment prime group: B = .16, t = 1.85, p = .067; slope for non-attachment prime group: B = .55, t = 5.46, p < .001).

Significant predictors of change in negative mood were again individualistic self-construal (B = .025, t = 4.09, p < .001), and individualistic self-construal in interaction with attachment prime group (B = -.025, t = -3.29, p = .001; final model F(3,88) = 5.91, p = .001, R^2^ = .17). Simple slopes analysis showed that in the attachment prime group, those with higher individualistic self-construal evidenced an increase in negative mood state relative to those with lower individualistic self-construal (slope: B = 0.025, t = 4.07, p = < .001).

### Discussion

This study aimed to examine the impact of attachment priming on the experience of social exclusion. Participants activated either an attachment or non-attachment social figure via imaginal priming prior to participating in the ‘Cyberball’ computerized ball tossing task to induce social exclusion. The key finding was that attachment priming had a significant influence on cardiovascular responding during and following exclusion. Specifically, we found that attachment priming was not associated with any significant change in HF-HRV during exclusion and recovery relative to baseline; whereas non-attachment priming was associated with a significant increase in HF-HRV during Cyberball followed by a decrement at recovery relative to baseline. Moreover, individual differences in rejection sensitivity, individualistic-collectivistic self-construal and anxious attachment style, significantly moderated the impact of the attachment prime on cardiovascular responses, as well as mood state changes. This was despite there being no differences in the subjective experience of the exclusion task or shifts in fundamental needs between the two prime groups. Overall, it appears that priming attachments could be an important buffer in maintaining cardiovagal homeostasis in the context of social exclusion, particularly amongst those who are less rejection sensitive and collectivistic in social orientation.

We hypothesized that the attachment prime would dampen sympathetic activations via heart rate, however we found exclusion to be associated with HR deceleration in general regardless of prime condition. The findings in this area are mixed. Some studies report HR acceleration to the Cyberball task in healthy subjects [10]. Another study in depressed participants found increased HR during the Cyberball amongst those with disorganized (or insecure) attachments compared to those with organized attachments [44]. However, there is also evidence to suggest HR decelerates in response to peer rejection, suggesting that unexpected exclusion can lead to the engagement of the parasympathetic nervous system and reduced sympathetic activation [45]. Moreover, HR deceleration is coupled with increases in HF-HRV; indeed both groups evidenced increases in HF-HRV during Cyberball relative to baseline, with the non-attachment prime group showing greater elevation–suggesting this group harnessed stronger self-regulation resources to better cope with social exclusion without the buffering activation of the attachment prime. However, at the expense of this increase, the non-attachment prime group demonstrated significant decline in HF-HRV relative to baseline in the recovery phase, suggesting that there is a cost physiological associated with the initial enhancement during exclusion itself.

Attachment theory suggests that attachment figures facilitate coping with adversity and stress [1, 19], and our findings suggest that this occurs via a conservation of cardiovascular resources. The attachment prime group showed relative stabilization of HF-HRV during exclusion and recovery compared to the non-attachment group–which was deprived of a supportive figure during imaginal priming. This finding is consistent with Social Baseline Theory, which argues proximity to social support acts to conserve physiological resources via load sharing [4]. Load sharing may lower the perceived threat of a social stressor, eliminating the need for excessive physiological responding [46]. This conservation of resources was reflected in HF-HRV in the current study, suggesting that activation of attachments may be a critical regulator of the Polyvagal system [14]. Neurocircuitry that governs HF-HRV also critically underpins emotion regulation and body-state feedback processes [47]. It is plausible that these neurobiological mechanisms are implicitly modulated by internalized human attachment systems, facilitating adaptive coping to social exclusion.

This study highlights the important role that individual differences play in moderating the influence of attachment priming during social exclusion, further extending attachment models. Highly rejection sensitive participants demonstrated comparatively stronger HR deceleration and HF-HRV increases during Cyberball when primed with an attachment figure, suggesting a hyper-vigilance to socially challenging situations [23] and more effortful attempts to self-regulate at a higher metabolic cost. Thus, rejection sensitivity may reduce the benefit afforded by attachment figures, thereby increasing the demand for personal resources in compensation when faced with a social stressor, thus aligning with Social Baseline Theory[4].

Self-construal also significantly influenced cardiovascular responses during social exclusion. If HR deceleration is associated with a heightened perception of exclusion, as the current results suggest, then attachment priming appears to exacerbate cardiovascular reactivity to exclusion in individualists, and dampened the response for collectivists. Consistent with this, stronger individualism was also associated with increases in negative mood state in the attachment prime group. Trait collectivists may benefit more from the implicit nature of the social support provided by attachment priming [48]. The findings also suggest that self-construal only affected the immediate experience of social exclusion, rather than the subsequent recovery of fundamental needs as previously demonstrated [29].

We found that high anxious attachment style influenced the experience of social exclusion by moderating HF-HRV: when primed with an attachment figure, HF-HRV did not fluctuate from baseline, compared to the non-attachment prime condition where those with anxious attachment style showed a trend towards elevated HF-HRV during the Cyberball. This suggests that anxiously attached individuals engaged stronger cardiovascular resources in the absence of the attachment prime to cope with social exclusion. Counter to our predictions, we did not observe any interaction of avoidant attachment style with attachment prime in terms of cardiovascular responses, however, positive mood was relatively stable following social exclusion in participants with greater avoidant insecurity in the non-attachment prime group. These findings suggest that avoidance was associated with greater benefit from the non-attachment prime in terms of positive affect, but not in heart rate.

In regards to limitations, we did not include a control condition without rejection in the current study. Given this was an extension of previous studies investigating the impact of attachments on the experience of exclusion and threat into the social domain, we were not interested in whether the buffering effects of imaginal attachment priming were specific to social exclusion. Future studies could include a non-rejection condition to examine specific vs general physiological responses to activating attachments via priming. We also did not observe strong effects of attachment insecurity on heart rate variables, which was unexpected given that many studies demonstrate that the effects of attachment priming is contingent on secure attachment style. This effect may be attributed to the characteristics of this participant cohort, who displayed only a moderate range of attachment insecurity scores. Future studies should examine these effects in insecurely attached populations or clinical cohorts to better examine the role of attachment insecurity. Further limitations of the study include the number of analyses conducted, particularly considering evidence that moderation analyses may be prone to Type 1 error [49], and a lack of a no-prime control group; which would assist in elucidating the specific effects of any social support in line with Social Baseline Theory. We also relied on subjective reports of explicitly measure affect. To circumvent potential demand characteristics, and to consider the role of implicit shifts in emotional state as a function of attachment priming in social exclusion, future studies could implement implicit measures of affect.

## Conclusions

Attachment figures may play a fundamental role in maintaining our social baseline in order to ensure optimal functioning, even in the face of social threat or pain. Findings from this study suggest that mentally activating attachment figures prior to experiencing temporary social exclusion has a significant buffering effect on cardiovascular reactions during and following exclusion—a finding that aligns with Social Baseline Theory. The effects of social exclusion can be powerful and aversive, with long-term detrimental effects on wellbeing and increased risk for psychopathology. Social attachments therefore may offer an internal system that can be harnessed to enhance protection against the psychosocial and physiological burden of socially isolating experiences.

## Supporting information

S1 File Supporting information presents tables of moderated hierarchical regression analyses–both initial models and final models, as described in the results. (DOCX)

S1 Dataset Dataset provides raw and aggregated data in an SPSS dataset used in the analyses described in the manuscript. (SAV)
