# Supplementary material for: Attachment buffers the physiological impact of social exclusion
Source: PLoS One. 2018 Sep 5;13(9):e0203287. doi: 10.1371/journal.pone.0203287 (PMC6124764; doi:10.1371/journal.pone.0203287)
Supplement: S1 File — (DOCX) [file pone.0203287.s001.docx]

**Supporting Information**

**S1 Table. Dependent variable – Change in heart rate during Cyberball**

*Coefficients for Moderated Hierarchical Regression Analysis: Initial Model*

| **Model** | **Unstandardised Coefficients** | | **Standardised Coefficients** |  |  |
| --- | --- | --- | --- | --- | --- |
|  | **B** | **Std. Error** | **β** | ***t*** | **Sig.** |
| **Step 1** |  |  |  |  |  |
| Constant | -.029 | .008 |  | -3.789 | .000* |
| Prime Condition | .005 | .011 | .046 | .435 | .664 |
| **Step 2** |  |  |  |  |  |
| Constant | -.030 | .008 |  | -3.831 | .000* |
| Prime Condition | .007 | .011 | .065 | .597 | .552 |
| Rejection sensitivity | -.001 | .002 | -.044 | -.337 | .737 |
| Individualistic self-construal | .000 | .001 | -.064 | -.475 | .636 |
| Collectivistic self-construal | .000 | .001 | .013 | 1.007 | .317 |
| Anxious attachment style | .012 | .011 | .131 | 1.007 | .317 |
| Avoidant attachment style | .003 | .008 | .046 | .375 | .708 |
| **Step 3** |  |  |  |  |  |
| Constant | -.031 | .007 |  | -4.163 | .000* |
| Prime Condition | .008 | .011 | .076 | .756 | .452 |
| Rejection sensitivity | -.005 | .003 | -.327 | -2.119 | .037* |
| Individualistic self-construal | -.003 | .001 | -.471 | -2.424 | .018* |
| Collectivistic self-construal | .002 | .001 | .383 | 2.211 | .030* |
| Anxious attachment style | .009 | .015 | .098 | .554 | .581 |
| Avoidant attachment style | .006 | .011 | .096 | .599 | .551 |
| Anxious attachment style x Prime Condition | .000 | .022 | .000 | .002 | .999 |
| Avoidant attachment style x Prime Condition | .002 | .016 | .015 | .096 | .924 |
| Rejection sensitivity x Prime Condition | .012 | .004 | .462 | 2.797 | .006* |
| Individualistic self construal x Prime Condition | .005 | .001 | .650 | 3.091 | .003* |
| Collectivistic self construal x Prime Condition | -.004 | .001 | -.465 | -2.669 | .009* |

*Note.* Dependent variable: Change in heart rate (HR) during Cyberball

** p* < .05

**S2 Table. Dependent variable – Change in heart rate during Cyberball**

*ANOVA Summary Table: Initial Model*

| **Model** | | **SS** | **df** | **MS** | **F** | **Sig.** |
| --- | --- | --- | --- | --- | --- | --- |
| 1 | Regression | .001 | 1 | .001 | .190 | .664^a^ |
|  | Residual | .255 | 90 | .003 |  |  |
|  | Total | .256 | 91 |  |  |  |
| 2 | Regression | .008 | 6 | .001 | .474 | .826^b^ |
|  | Residual | .247 | 85 | .003 |  |  |
|  | Total | .256 | 91 |  |  |  |
| 3 | Regression | .054 | 11 | .005 | 1.959 | .044*^c^ |
|  | Residual | .206 | 84 | .002 |  |  |
|  | Total | .256 | 91 |  |  |  |

*Note.* Dependent variable: Change in HR during Cyberball

^a^ Predictors: Constant, Condition

^b^ Predictors: Constant, Condition, Collectivistic self-construal, Avoidant attachment style, anxious attachment style, Rejection sensitivity, Individualistic self-construal

^c^ Predictors: Constant, Condition, Collectivistic self-construal, Avoidant attachment style, Anxious attachment style, Rejection sensitivity, Individualistic self-construal, Rejection sensitivity x Condition, Avoidant attachment style x Condition, Anxious attachment style x Condition, Collectivistic self-construal x Condition, Individualistic self-construal x Condition

* *p* < .05

**S3 Table. Dependent variable – Change in heart rate during Cyberball**

*Coefficients for Moderated Hierarchical Regression Analysis: Final Model*

| **Model** | **Unstandardised Coefficients** | | **Standardised Coefficients** |  |  |
| --- | --- | --- | --- | --- | --- |
|  | **B** | **Std. Error** | **β** | ***t*** | **Sig.** |
| **Step 1** |  |  |  |  |  |
| Constant | -.029 | .008 |  | -3.789 | .000* |
| Prime Condition | .005 | .011 | .046 | .435 | .664 |
| **Step 2** |  |  |  |  |  |
| Constant | -.030 | .008 |  | -3.831 | .000* |
| Prime Condition | .006 | .011 | .061 | .568 | .571 |
| Rejection sensitivity | .000 | .002 | .003 | .021 | .983 |
| Individualistic self-construal | -.001 | .001 | -.124 | -1.011 | .315 |
| Collectivistic self-construal | .000 | .001 | .034 | .316 | .753 |
| **Step 3** |  |  |  |  |  |
| Constant | -.031 | .007 |  | -4.218 | .000 |
| Prime Condition | .008 | .010 | .075 | .757 | .451 |
| Rejection sensitivity | -.004 | .002 | -.273 | -1.915 | .059 |
| Individualistic self-construal | -.003 | .001 | -.535 | -3.036 | .003* |
| Collectivistic self-construal | .002 | .001 | .386 | 2.296 | .024* |
| Rejection sensitivity x Prime Condition | .011 | .003 | .459 | 2.999 | .004* |
| Individualistic self-construal x Prime Condition | .005 | .001 | .642 | 3.439 | .001* |
| Collectivistic self-construal x Prime Condition | -.004 | .001 | -.450 | -2.680 | .009* |

* *p* < .05

**S4 Table. Dependent variable – Change in heart rate during Cyberball** *ANOVA Summary Table: Final Model*

| **Model** | | **SS** | **df** | **MS** | **F** | **Sig.** |
| --- | --- | --- | --- | --- | --- | --- |
| 1 | Regression | .001 | 1 | .001 | .190 | .664^a^ |
|  | Residual | .255 | 90 | .003 |  |  |
|  | Total | .256 | 91 |  |  |  |
| 2 | Regression | .005 | 4 | .001 | .391 | .815^b^ |
|  | Residual | .251 | 87 | .003 |  |  |
|  | Total | .256 | 91 |  |  |  |
| 3 | Regression | .050 | 7 | .007 | 2.908 | .009*^c^ |
|  | Residual | .206 | 84 | .002 |  |  |
|  | Total | .256 | 91 |  |  |  |

*Note.* Dependent variable: Change in HR during Cyberball

(Table of coefficients is provided in body of thesis (Table 5))

^a^ Predictors: Constant, Condition

^b^ Predictors: Constant, Condition, Collectivistic self-construal, Rejection sensitivity, Individualistic self-construal

^c^ Predictors: Constant, Condition, Collectivistic self-construal, Rejection sensitivity, Individualistic self-construal, Rejection sensitivity x Condition, Collectivistic self-construal x Condition, Individualistic self-construal x Condition

* *p* < .05

**S5 Table. Dependent variable – Change in heart rate during recovery**

*Coefficients for Moderated Hierarchical Regression Analysis: Initial Model*

| **Model** | **Unstandardised Coefficients** | | **Standardised Coefficients** |  |  |
| --- | --- | --- | --- | --- | --- |
|  | **B** | **Std. Error** | **β** | ***t*** | **Sig.** |
| **Step 1** |  |  |  |  |  |
| Constant | -.004 | .007 |  | -.555 | .580 |
| Prime Condition | .012 | .010 | .130 | 1.239 | .218 |
| **Step 2** |  |  |  |  |  |
| Constant | -.004 | .007 |  | -.523 | .603 |
| Condition | .012 | .010 | .127 | 1.173 | .244 |
| Rejection sensitivity | -.001 | .002 | -.102 | -.784 | .435 |
| Individualistic self-construal | .000 | .001 | -.047 | -.352 | .726 |
| Collectivistic self-construal | .000 | .001 | -.074 | -.669 | .505 |
| Anxious attachment style | .004 | .010 | .047 | .365 | .716 |
| Avoidant attachment style | .004 | .007 | .063 | .514 | .609 |
| **Step 3** |  |  |  |  |  |
| Constant | -.003 | .007 |  | -.385 | .701 |
| Condition | .012 | .010 | .131 | 1.198 | .234 |
| Rejection sensitivity | -.003 | .002 | -.212 | -1.268 | .209 |
| Individualistic self-construal | -.001 | .001 | -.131 | -.623 | .535 |
| Collectivistic self-construal | .000 | .001 | -.030 | -.161 | .873 |
| Anxious attachment style | -.005 | .015 | -.068 | -.358 | .721 |
| Avoidant attachment style | .003 | .010 | .057 | .327 | .744 |
| Anxious attachment style x Prime Condition | .019 | .021 | .167 | .892 | .375 |
| Avoidant attachment style x Prime Condition | .006 | .015 | .069 | .393 | .695 |
| Rejection sensitivity x Prime Condition | .004 | .004 | .191 | 1.069 | .288 |
| Individualistic self-construal x Prime Condition | .001 | .001 | .200 | .879 | .382 |
| Collectivistic self-construal x Prime Condition | .000 | .001 | -.065 | -.345 | .731 |

*Note.* Dependent variable: Change in HR during Recovery

* *p* < .05

**S6 Table. Dependent variable – Change in heart rate during recovery**

*ANOVA Summary Table: Initial Model*

| **Model** | | **SS** | **df** | **MS** | **F** | **Sig.** |
| --- | --- | --- | --- | --- | --- | --- |
| 1 | Regression | .003 | 1 | .003 | 1.536 | .218^a^ |
|  | Residual | .196 | 90 | .002 |  |  |
|  | Total | .199 | 91 |  |  |  |
| 2 | Regression | .007 | 6 | .001 | .514 | .796^b^ |
|  | Residual | .192 | 85 | .002 |  |  |
|  | Total | .199 | 91 |  |  |  |
| 3 | Regression | .015 | 11 | .001 | .573 | .846^c^ |
|  | Residual | .185 | 80 | .002 |  |  |
|  | Total | .199 | 91 |  |  |  |

*Note.* Dependent variable: Change in HR during Recovery

^a^ Predictors: Constant, Condition

^b^ Predictors: Constant, Condition, Collectivistic self-construal, Avoidant attachment style, Anxious attachment style, Rejection sensitivity, Individualistic self-construal

^c^ Predictors: Constant, Condition, Collectivistic self-construal, Avoidant attachment style, Anxious attachment style, Rejection sensitivity, Individualistic self-construal, Rejection sensitivity x Condition, Avoidant attachment style x Condition, Anxious attachment style x Condition, Collectivistic self-construal x Condition, Individualistic self-construal x Condition

* *p* < .05

**S7 Table. Dependent variable – Change in HF(nu) during Cyberball**

*Coefficients for Moderated Hierarchical Regression Analysis: Initial Model*

| **Model** | **Unstandardised Coefficients** | | **Standardised Coefficients** |  |  |
| --- | --- | --- | --- | --- | --- |
|  | **B** | **Std. Error** | **β** | ***t*** | **Sig.** |
| **Step 1** |  |  |  |  |  |
| Constant | 1.578 | 2.512 |  | .628 | .531 |
| Prime Condition | 3.619 | 3.591 | .106 | 1.008 | .316 |
| **Step 2** |  |  |  |  |  |
| Constant | 1.355 | 2.554 |  | .531 | .597 |
| Prime Condition | 4.074 | 3.678 | .119 | 1.108 | .271 |
| Rejection sensitivity | 1.055 | .682 | .200 | 1.546 | .126 |
| Individualistic self-construal | .309 | .245 | .169 | 1.263 | .210 |
| Collectivistic self-construal | -.018 | .228 | -.009 | -.078 | .938 |
| Anxious attachment style | .703 | .3681 | .025 | .191 | .849 |
| Avoidant attachment style | -.125 | 2.652 | -.006 | -.047 | .963 |
| **Step 3** |  |  |  |  |  |
| Constant | 1.609 | 2.494 |  | .645 | .521 |
| Prime Condition | 3.740 | 3.567 | .109 | 1.049 | .298 |
| Rejection sensitivity | 2.222 | .841 | .422 | 2.642 | .010* |
| Individualistic self-construal | .699 | .368 | .382 | 1.899 | .061 |
| Collectivistic self-construal | -.156 | .370 | -.076 | -.422 | .674 |
| Anxious attachment style | -5.176 | 5.201 | -.181 | -.995 | .323 |
| Avoidant attachment style | .500 | 3.609 | .023 | .139 | .890 |
| Anxious attachment style x Prime Condition | 13.697 | 7.337 | .333 | 1.867 | .066 |
| Avoidant attachment style x Prime Condition | -.858 | 5.320 | -.027 | -.161 | .872 |
| Rejection sensitivity x Prime Condition | -3.311 | 1.381 | -.409 | -2.398 | .019* |
| Individualistic self-construal x Prime Condition | -.786 | .500 | -.341 | -1.571 | .120 |
| Collectivistic self-construal x Prime Condition | .168 | .463 | .065 | .362 | .718 |

*Note.* Dependent variable: Change in HF(nu) during Cyberball

* *p* < .05

**S8 Table. Dependent variable – Change in HF(nu) during Cyberball**

*ANOVA Summary Table: Initial Model*

| **Model** | | **SS** | **df** | **MS** | **F** | **Sig.** |
| --- | --- | --- | --- | --- | --- | --- |
| 1 | Regression | 301.127 | 1 | 301.127 | 1.016 | .316^a^ |
|  | Residual | 26686.9 | 90 | 296.521 |  |  |
|  | Total | 269988.0 | 91 |  |  |  |
| 2 | Regression | 1293.33 | 6 | 215.556 | .713 | .713^b^ |
|  | Residual | 25694.7 | 85 | 302.290 |  |  |
|  | Total | 26988.0 | 91 |  |  |  |
| 3 | Regression | 4282.48 | 11 | 389.317 | 1.372 | .203^c^ |
|  | Residual | .206 | 84 | .002 |  |  |
|  | Total | .256 | 91 |  |  |  |

*Note.* Dependent variable: Change in HF(nu) during Cyberball

^a^ Predictors: Constant, Condition

^b^ Predictors: Constant, Condition, Collectivistic self-construal, Avoidant attachment style, Anxious attachment style, Rejection sensitivity, Individualistic self-construal

^c^ Predictors: Constant, Condition, Collectivistic self-construal, Avoidant attachment style, Anxious attachment style, Rejection sensitivity, Individualistic self-construal, Rejection sensitivity x Condition, Avoidant attachment style x Condition, Anxious attachment style x Condition, Collectivistic self-construal x Condition, Individualistic self-construal x Condition

* *p* < .05

**S9 Table. Dependent variable – Change in HF(nu) during Cyberball**

*Coefficients for Moderated Hierarchical Regression Analysis: Final Model*

| **Model** | **Unstandardised Coefficients** | | **Standardised Coefficients** |  |  |
| --- | --- | --- | --- | --- | --- |
|  | **B** | **Std. Error** | **β** | ***t*** | **Sig.** |
| **Step 1** |  |  |  |  |  |
| Constant | 1.578 | 2.512 |  | .628 | .531 |
| Prime Condition | 3.619 | 3.591 | .106 | 1.008 | .316 |
| **Step 2** |  |  |  |  |  |
| Constant | 1.356 | 2.524 |  | .537 | .592 |
| Prime Condition | 4.073 | 3.634 | .119 | 1.121 | .265 |
| Rejection sensitivity | 1.044 | .657 | .198 | 1.589 | .116 |
| Anxious attachment style | .623 | 3.527 | .022 | .177 | .860 |
| Individualistic self-construal | .307 | .230 | .168 | 1.337 | .185 |
| **Step 3** |  |  |  |  |  |
| Constant | 1.377 | 2.455 |  | .561 | .576 |
| Prime Condition | 3.978 | 3.514 | .116 | 1.132 | .261 |
| Rejection sensitivity | 2.000 | .784 | .380 | 2.552 | .012* |
| Anxious attachment style | -7.458 | 4.569 | -.261 | -1.632 | .106 |
| Individualistic self-construal | .232 | .229 | .126 | 1.013 | .314 |
| Anxious attachment style x Prime Condition | 17.290 | 6.509 | .420 | 2.656 | .009* |
| Rejection sensitivity x Prime Condition | -2.596 | 1.254 | -.321 | -2.070 | .041* |

* *p* < .05

**S10 Table. Dependent variable – Change in HF(nu) during Cyberball**

*ANOVA Summary Table: Final Model*

| **Model** | | **SS** | **df** | **MS** | **F** | **Sig.** |
| --- | --- | --- | --- | --- | --- | --- |
| 1 | Regression | 301.127 | 1 | 301.127 | 1.016 | .316^a^ |
|  | Residual | 26686.9 | 90 | 296.521 |  |  |
|  | Total | 269988.0 | 91 |  |  |  |
| 2 | Regression | 1290.92 | 4 | 322.731 | 1.093 | .365^b^ |
|  | Residual | 25697.1 | 87 | 295.369 |  |  |
|  | Total | 26988.0 | 91 |  |  |  |
| 3 | Regression | 3513.02 | 6 | 585.503 | 2.120 | .059^c^ |
|  | Residual | .206 | 84 | .002 |  |  |
|  | Total | .256 | 91 |  |  |  |

*Note.* Dependent variable: Change in HF(nu) during Cyberball

^a^ Predictors: Constant, Condition

^b^ Predictors: Constant, Condition, Individualistic self-construal, Anxious attachment style, Rejection sensitivity

^c^ Predictors: Constant, Condition, Individualistic self-construal, Anxious attachment style, Rejection sensitivity, Rejection sensitivity x Condition, Anxious attachment style x Condition

* *p* < .05

**S11 Table. Dependent variable – Change in HF(nu) during recovery**

*Coefficients for Moderated Hierarchical Regression Analysis: Initial Model*

| **Model** | **Unstandardised Coefficients** | | **Standardised Coefficients** |  |  |
| --- | --- | --- | --- | --- | --- |
|  | **B** | **Std. Error** | **β** | ***t*** | **Sig.** |
| **Step 1** |  |  |  |  |  |
| Constant | -.299 | 2.234 |  | -.129 | .898 |
| Prime Condition | -2.664 | 3.324 | -.084 | -.801 | .423 |
| **Step 2** |  |  |  |  |  |
| Constant | -.339 | 2.334 |  | -.145 | .885 |
| Prime Condition | -2.582 | 3.362 | -.082 | -.768 | .445 |
| Rejection sensitivity | .794 | .624 | .163 | 1.273 | .206 |
| Individualistic self-construal | .097 | .224 | .058 | .435 | .665 |
| Collectivistic self-construal | .328 | .208 | .172 | 1.578 | .118 |
| Anxious attachment style | -3.785 | 3.364 | -.144 | -1.125 | .264 |
| Avoidant attachment style | -.847 | 2.424 | -.042 | -.349 | .728 |
| **Step 3** |  |  |  |  |  |
| Constant | -.020 | 2.358 |  | -.009 | .993 |
| Prime Condition | -2.742 | 3.371 | -.087 | -.813 | .418 |
| Rejection sensitivity | 1.283 | .795 | .264 | 1.614 | .110 |
| Individualistic self-construal | .401 | .348 | .237 | 1.151 | .253 |
| Collectivistic self-construal | .249 | .349 | .130 | .711 | .479 |
| Anxious attachment style | -5.992 | 4.916 | -.227 | -1.219 | .226 |
| Avoidant attachment style | -2.603 | 3.411 | -.129 | -.763 | .448 |
| Anxious attachment style x Prime Condition | 6.908 | 6.935 | .182 | .996 | .322 |
| Avoidant attachment style x Prime Condition | 4.191 | 5.029 | .142 | .833 | .407 |
| Rejection sensitivity x Prime Condition | -1.334 | 1.306 | -.178 | -1.022 | .310 |
| Individualistic self-construal x Prime Condition | -.433 | .473 | -.203 | -.915 | .363 |
| Collectivistic self-construal x Prime Condition | .084 | .438 | .035 | .192 | .848 |

*Note.* Dependent variable: Change in HF(nu) during Recovery

* *p* < .05

**S12 Table. Dependent variable – Change in HF(nu) during recovery**

*ANOVA Summary Table: Initial Model*

| **Model** | | **SS** | **df** | **MS** | **F** | **Sig.** |
| --- | --- | --- | --- | --- | --- | --- |
| 1 | Regression | 163.099 | 1 | 163.099 | .642 | .425^a^ |
|  | Residual | 22853.9 | 90 | 243.932 |  |  |
|  | Total | 23017.0 | 91 |  |  |  |
| 2 | Regression | 1548.79 | 6 | 258.131 | 1.022 | .417^b^ |
|  | Residual | 21468.2 | 85 | 252.567 |  |  |
|  | Total | 23017.0 | 91 |  |  |  |
| 3 | Regression | 2727.69 | 11 | 247.972 | .978 | .473^c^ |
|  | Residual | 20289.3 | 80 | 253.616 |  |  |
|  | Total | 23017.0 | 91 |  |  |  |

*Note.* Dependent variable: Change in HF(nu) during Recovery

^a^ Predictors: Constant, Condition

^b^ Predictors: Constant, Condition, Collectivistic self-construal, Avoidant attachment style, Anxious attachment style, Rejection sensitivity, Individualistic self-construal

^c^ Predictors: Constant, Condition, Collectivistic self-construal, Avoidant attachment style, Anxious attachment style, Rejection sensitivity, Individualistic self-construal, Rejection sensitivity x Condition, Avoidant attachment style x Condition, Anxious attachment style x Condition, Collectivistic self-construal x Condition, Individualistic self-construal x Condition

* *p* < .05

**S13 Table. Dependent variable – Change in positive mood**

*Coefficients for Moderated Hierarchical Regression Analysis: Initial Model*

| **Model** | **Unstandardised Coefficients** | | **Standardised Coefficients** |  |  |
| --- | --- | --- | --- | --- | --- |
|  | **B** | **Std. Error** | **β** | ***t*** | **Sig.** |
| **Step 1** |  |  |  |  |  |
| Constant | -0.409 | 0.072 |  | -5.693 | 0.000 |
| Prime Condition | 0.173 | 0.103 | 0.175 | 1.686 | 0.095 |
| **Step 2** |  |  |  |  |  |
| Constant | -0.387 | 0.071 |  | -5.473 | 0.000 |
| Prime Condition | 0.149 | 0.101 | 0.151 | 1.469 | 0.145 |
| Rejection sensitivity | -0.013 | 0.019 | -0.084 | -0.680 | 0.499 |
| Individualistic self-construal | 0.015 | 0.007 | 0.278 | 2.180 | 0.032* |
| Collectivistic self-construal | -0.001 | 0.006 | -0.020 | -0.188 | 0.851 |
| Anxious attachment style | 0.039 | 0.101 | 0.047 | 0.383 | 0.703 |
| Avoidant attachment style | 0.185 | 0.073 | 0.293 | 2.528 | 0.013* |
| **Step 3** |  |  |  |  |  |
| Constant | -0.375 | 0.069 |  | -5.418 | 0.000 |
| Prime Condition | 0.150 | 0.098 | 0.151 | 1.526 | 0.131 |
| Rejection sensitivity | -0.002 | 0.023 | -0.014 | -0.094 | 0.925 |
| Individualistic self-construal | 0.024 | 0.010 | 0.458 | 2.401 | 0.019* |
| Collectivistic self-construal | -0.009 | 0.010 | -0.144 | -0.845 | 0.401 |
| Anxious attachment style | 0.072 | 0.143 | 0.087 | 0.505 | 0.615 |
| Avoidant attachment style | 0.003 | 0.099 | 0.004 | 0.028 | 0.978 |
| Anxious attachment style x Prime Condition | 0.072 | 0.201 | 0.060 | 0.357 | 0.722 |
| Avoidant attachment style x Prime Condition | 0.398 | 0.146 | 0.433 | 2.730 | 0.008* |
| Rejection sensitivity x Prime Condition | -0.020 | 0.038 | -0.084 | -0.521 | 0.604 |
| Individualistic self construal x Prime Condition | -0.007 | 0.014 | -0.105 | -0.510 | 0.612 |
| Collectivistic self construal x Prime Condition | 0.009 | 0.013 | 0.127 | 0.740 | 0.461 |

*Note.* Dependent variable: Change in Positive mood (PANAS)

** p* < .05

**S14 Table. Dependent variable – Change in positive mood**

*ANOVA Summary Table: Initial Model*

| **Model** | | **SS** | **df** | **MS** | **F** | **Sig.** |
| --- | --- | --- | --- | --- | --- | --- |
| 1 | Regression | 0.688 | 1 | 0.688 | 2.842 | .095^a^ |
|  | Residual | 21.780 | 90 | 0.242 |  |  |
|  | Total | 22.467 | 91 |  |  |  |
| 2 | Regression | 2.954 | 6 | 0.492 | 2.145 | .056^b^ |
|  | Residual | 19.513 | 85 | 0.230 |  |  |
|  | Total | 22.467 | 91 |  |  |  |
| 3 | Regression | 5.402 | 11 | 0.491 | 2.302 | .017^c^* |
|  | Residual | 17.065 | 80 | 0.213 |  |  |
|  | Total | 22.467 | 91 |  |  |  |

*Note.* Dependent variable: Change i

^a^ Predictors: Constant, Condition

^b^ Predictors: Constant, Condition, Collectivistic self-construal, Avoidant attachment style, Anxious attachment style, Rejection sensitivity, Individualistic self-construal

^c^ Predictors: Constant, Condition, Collectivistic self-construal, Avoidant attachment style, Anxious attachment style, Rejection sensitivity, Individualistic self-construal, Rejection sensitivity x Condition, Avoidant attachment style x Condition, Anxious attachment style x Condition, Collectivistic self-construal x Condition, Individualistic self-construal x Condition

* *p* < .05

**S15 Table. Dependent variable – Change in positive mood**

*Coefficients for Moderated Hierarchical Regression Analysis: Final Model*

| **Model** | **Unstandardised Coefficients** | | **Standardised Coefficients** |  |  |
| --- | --- | --- | --- | --- | --- |
|  | **B** | **Std. Error** | **β** | ***t*** | **Sig.** |
| **Step 1** |  |  |  |  |  |
| Constant | -0.409 | 0.072 |  | -5.693 | 0.000 |
| Prime Condition | 0.173 | 0.103 | 0.175 | 1.686 | 0.095 |
| **Step 2** |  |  |  |  |  |
| Constant | -0.392 | 0.069 |  | -5.647 | 0.000 |
| Prime Condition | 0.155 | 0.099 | 0.156 | 1.559 | 0.122 |
| Individualistic self-construal | 0.015 | 0.006 | 0.288 | 2.621 | 0.010 |
| Avoidant attachment style | 0.177 | 0.069 | 0.282 | 2.575 | 0.012 |
| **Step 3** |  |  |  |  |  |
| Constant | -0.382 | 0.066 |  | -5.787 | 0.000 |
| Prime Condition | 0.155 | 0.094 | 0.157 | 1.644 | 0.104 |
| Individualistic self-construal | 0.019 | 0.006 | 0.360 | 3.364 | 0.001 |
| Avoidant attachment style | 0.013 | 0.084 | 0.020 | 0.150 | 0.881 |
| Avoidant attachment style x Prime Condition | 0.391 | 0.123 | 0.425 | 3.191 | 0.002 |

*Note.* Dependent variable: Change in Positive mood (PANAS)

** p* < .05

**S16 Table. Dependent variable – Change in positive mood**

*ANOVA Summary Table: Final Model*

| **Model** | | **SS** | **df** | **MS** | **F** | **Sig.** |
| --- | --- | --- | --- | --- | --- | --- |
| 1 | Regression | 0.688 | 1 | 0.688 | 2.842 | .095^a^ |
|  | Residual | 21.780 | 90 | 0.242 |  |  |
|  | Total | 22.467 | 91 |  |  |  |
| 2 | Regression | 2.826 | 3 | 0.942 | 4.221 | .008^b^* |
|  | Residual | 19.641 | 88 | 0.223 |  |  |
|  | Total | 22.467 | 91 |  |  |  |
| 3 | Regression | 4.884 | 4 | 1.221 | 6.041 | .000^c^* |
|  | Residual | 17.584 | 87 | 0.202 |  |  |
|  | Total | 22.467 | 91 |  |  |  |

*Note.* Dependent variable: Change in positive mood

^a^ Predictors: Constant, Condition

^b^ Predictors: Constant, Condition, Avoidant attachment style, Individualistic self-construal

^c^ Predictors: Constant, Condition, Avoidant attachment style, Individualistic self-construal, Avoidant attachment style x Condition

* *p* < .05

**S17 Table. Dependent variable – Change in negative mood**

*Coefficients for Moderated Hierarchical Regression Analysis: Initial Model*

| **Model** | **Unstandardised Coefficients** | | **Standardised Coefficients** |  |  |
| --- | --- | --- | --- | --- | --- |
|  | **B** | **Std. Error** | **β** | ***t*** | **Sig.** |
| **Step 1** |  |  |  |  |  |
| Constant | -0.140 | 0.052 |  | -2.707 | 0.008 |
| Prime Condition | 0.069 | 0.074 | 0.098 | 0.934 | 0.353 |
| **Step 2** |  |  |  |  |  |
| Constant | -0.127 | 0.052 |  | -2.438 | 0.017 |
| Prime Condition | 0.053 | 0.075 | 0.075 | 0.706 | 0.482 |
| Rejection sensitivity | 0.001 | 0.014 | 0.006 | 0.049 | 0.961 |
| Individualistic self-construal | 0.012 | 0.005 | 0.320 | 2.432 | 0.017* |
| Collectivistic self-construal | -0.003 | 0.005 | -0.065 | -0.605 | 0.547 |
| Anxious attachment style | 0.046 | 0.075 | 0.078 | 0.615 | 0.540 |
| Avoidant attachment style | 0.038 | 0.054 | 0.084 | 0.705 | 0.483 |
| **Step 3** |  |  |  |  |  |
| Constant | -0.109 | 0.050 |  | -2.171 | 0.033 |
| Prime Condition | 0.045 | 0.071 | 0.064 | 0.636 | 0.527 |
| Rejection sensitivity | -0.006 | 0.017 | -0.052 | -0.336 | 0.738 |
| Individualistic self-construal | 0.027 | 0.007 | 0.722 | 3.727 | 0.000* |
| Collectivistic self-construal | 0.004 | 0.007 | 0.100 | 0.579 | 0.564 |
| Anxious attachment style | 0.117 | 0.103 | 0.199 | 1.135 | 0.260 |
| Avoidant attachment style | -0.035 | 0.072 | -0.077 | -0.485 | 0.629 |
| Anxious attachment style x Prime Condition | -0.060 | 0.146 | -0.071 | -0.413 | 0.681 |
| Avoidant attachment style x Prime Condition | 0.140 | 0.106 | 0.213 | 1.324 | 0.189 |
| Rejection sensitivity x Prime Condition | 0.012 | 0.027 | 0.070 | 0.430 | 0.669 |
| Individualistic self-construal x Prime Condition | -0.020 | 0.010 | -0.419 | -2.008 | 0.048* |
| Collectivistic self-construal x Prime Condition | -0.011 | 0.009 | -0.199 | -1.144 | 0.256 |

*Note.* Dependent variable: Change in Negative mood (PANAS)

** p* < .05

**S18 Table. Dependent variable – Change in negative mood**

*ANOVA Summary Table: Initial Model*

| **Model** | | **SS** | **df** | **MS** | **F** | **Sig.** |
| --- | --- | --- | --- | --- | --- | --- |
| 1 | Regression | 0.110 | 1 | 0.110 | 0.873 | .353^a^ |
|  | Residual | 11.386 | 90 | 0.127 |  |  |
|  | Total | 11.496 | 91 |  |  |  |
| 2 | Regression | 0.924 | 6 | 0.154 | 1.238 | .295^b^ |
|  | Residual | 10.573 | 85 | 0.124 |  |  |
|  | Total | 11.496 | 91 |  |  |  |
| 3 | Regression | 2.517 | 11 | 0.229 | 2.039 | .035^c^ |
|  | Residual | 8.979 | 80 | 0.112 |  |  |
|  | Total | 11.496 | 91 |  |  |  |

*Note.* Dependent variable: Change in Negative Mood

^a^ Predictors: Constant, Condition

^b^ Predictors: Constant, Condition, Collectivistic self-construal, Avoidant attachment style, Anxious attachment style, Rejection sensitivity, Individualistic self-construal

^c^ Predictors: Constant, Condition, Collectivistic self-construal, Avoidant attachment style, Anxious attachment style, Rejection sensitivity, Individualistic self-construal, Rejection sensitivity x Condition, Avoidant attachment style x Condition, Anxious attachment style x Condition, Collectivistic self-construal x Condition, Individualistic self-construal x Condition

* *p* < .05

**S19 Table. Dependent variable – Change in negative mood**

*Coefficients for Moderated Hierarchical Regression Analysis: Final Model*

| **Model** | **Unstandardised Coefficients** | | **Standardised Coefficients** |  |  |
| --- | --- | --- | --- | --- | --- |
|  | **B** | **Std. Error** | **β** | ***t*** | **Sig.** |
| **Step 1** |  |  |  |  |  |
| Constant | -0.140 | 0.052 |  | -2.707 | 0.008 |
| Prime Condition | 0.069 | 0.074 | 0.098 | 0.934 | 0.353 |
| **Step 2** |  |  |  |  |  |
| Constant | -0.128 | 0.051 |  | -2.508 | 0.014 |
| Prime Condition | 0.050 | 0.073 | 0.071 | 0.687 | 0.494 |
| Individualistic self-construal | 0.009 | 0.004 | 0.237 | 2.300 | 0.024* |
| **Step 3** |  |  |  |  |  |
| Constant | -0.105 | 0.049 |  | -2.153 | 0.034 |
| Prime Condition | 0.034 | 0.069 | 0.048 | 0.494 | 0.623 |
| Individualistic self-construal | 0.025 | 0.006 | 0.664 | 4.087 | 0.000* |
| Individualistic self-construal x Prime Condition | -0.025 | 0.008 | -0.532 | -3.291 | 0.001* |

*Note.* Dependent variable: Change in Negative mood (PANAS)

** p* < .05

**S20 Table. Dependent variable – Change in negative mood**

*ANOVA Summary Table: Final Model*

| **Model** | | **SS** | **df** | **MS** | **F** | **Sig.** |
| --- | --- | --- | --- | --- | --- | --- |
| 1 | Regression | 0.110 | 1 | 0.110 | 0.873 | .353^a^ |
|  | Residual | 11.386 | 90 | 0.127 |  |  |
|  | Total | 11.496 | 91 |  |  |  |
| 2 | Regression | 0.749 | 2 | 0.375 | 3.102 | .050^b^ |
|  | Residual | 10.747 | 89 | 0.121 |  |  |
|  | Total | 11.496 | 91 |  |  |  |
| 3 | Regression | 1.927 | 3 | 0.642 | 5.907 | .001^c^* |
|  | Residual | 9.569 | 88 | 0.109 |  |  |
|  | Total | 11.496 | 91 |  |  |  |

*Note.* Dependent variable: Change in Negative Mood

^a^ Predictors: Constant, Condition

^b^ Predictors: Constant, Condition, Individualistic self-construal

^c^ Predictors: Constant, Condition, Individualistic self-construal, Individualistic self-construal x Condition

* *p* < .05
